# *Lactobacillus reuteri* DSM 17938 Changes the Frequency of Foxp3^+^ Regulatory T Cells in the Intestine and Mesenteric Lymph Node in Experimental Necrotizing Enterocolitis

**DOI:** 10.1371/journal.pone.0056547

**Published:** 2013-02-20

**Authors:** Yuying Liu, Nicole Y. Fatheree, Bridgette M. Dingle, Dat Q. Tran, Jon Marc Rhoads

**Affiliations:** Divisions of Gastroenterology and Allergy-Immunology, Department of Pediatrics, The University of Texas Health Science Center at Houston Medical School, Houston, Texas, United States of America; Centro di Riferimento Oncologico, IRCCS National Cancer Institute, Italy

## Abstract

Necrotizing enterocolitis (NEC) is an inflammatory disease of the intestine in premature infants. *Lactobacillus reuteri* DSM 17938 improves survival and reduces the incidence and severity of NEC in a rodent model. Foxp3^+^ regulatory T cells (Tregs) maintain intestinal homeostasis by controlling inflammation and inducing tolerance. To determine whether there are insufficient numbers of Tregs to control inflammation in NEC and to determine if LR17938 increases the frequency of Tregs, we studied selected groups of newborn Sprague-Dawley rats according to feeding plan: dam±LR17938, formula±LR17938, and NEC±LR17938. NEC was induced by gavage feeding with special formula and exposure to hypoxic conditions. Lymphocytes isolated from ileum, mesenteric lymph nodes (MLN), spleen and thymus were labeled for T cell surface markers (CD3, CD4, CD8) and intracellular Foxp3; and labeled cells were analyzed by flow cytometry. The percentage of CD3^+^ T cells and Foxp3^+^ Tregs in the ileum significantly decreased in pups with NEC, compared to normal controls. Feeding LR17938 to neonatal rats with NEC increased the % of Foxp3^+^ T cells in the ileum while decreasing the percentage of cells in the MLN. Administration of LR17938 to dam-fed rats significantly increased Foxp3^+^Tregs in the ileum as early as day of life (DOL)1 but did not produce an increase in Tregs in formula-fed rats on DOL1. These results suggest that factors in breast milk may enhance the early immunomodulatory effects of LR17938. An anti-inflammatory effect of LR17938 in NEC was associated with the modulation of immune responses and induction and what appears to be migration of Foxp3^+^ Tregs to the diseased gut. Probiotic-facilitated development of Tregs might play an important role in the prevention of NEC.

## Introduction

Necrotizing enterocolitis (NEC), the leading cause of intestinal disease and short bowel syndrome in neonates, has become a priority for translational research [1]. While the pathophysiology of NEC is incompletely understood, research suggests that preterm infants have reduced fecal microbial diversity, which may impair the normal evolution of GI mucosal immune response [2]. To maintain intestinal health, the immune system must robustly respond to antigens from pathogenic microbes while controlling any reaction to self-molecules and commensals.

The intestinal mucosa contains gut-associated lymphoid tissue (GALT), the largest immunologic tissue in the body, which deals with these luminal antigens. Anatomically, GALT consists of organized lymphoid structures and diffuse populations of cells. Organized GALT comprises Peyer’s patches, isolated follicles, and mesenteric lymph nodes (MLN). Diffuse GALT consists of two distinct populations above and below the basement membrane, respectively: the lumen-facing intraepithelial lymphocytes (IEL) and the lamina propria lymphocytes (LPL). Both diffuse compartments contain mature T lymphocytes, but their biological functions are not well understood. T cells comprise both γδ and αβ T cells, the latter subset predominating in most species including the rat [3]. Functional definition of T subsets conventionally includes helper T (CD3^+^CD4^+^), cytotoxic T (CD3^+^CD8^+^), and regulatory T (“Treg,” CD3^+^CD4^+^CD25^+^ Foxp3^+^) T cells [4]. An undifferentiated CD4-positive T helper cell becomes activated and differentiates into one of four various subtypes, Th1, Th2, Th17 and Treg, following stimulation by cytokines. Among them, only Tregs regulate and attenuate all other T helper subtypes to induce self-tolerance and down-regulation following an acute insult. The expression of the transcription factor Foxp3 is required for Treg generation and maintenance. Although the validity of the Foxp3 marker to define human Treg is imperfect, it remains the most commonly used and unambiguous marker available to identify Treg in rodents and humans [5].

Preterm infants when compared to term infants have different postnatal gastrointestinal exposures which may lead to abnormal gut bacterial colonization and development [6]. Preterm infants are usually nursed in high-sanitary incubators with restricted breast feeding and human contact which may favor colonization with environmental bacteria and increase the risk of bacterial overgrowth [7]. *Bifidobacteria* and *Lactobacilli* are present in lower densities compared with term breast-fed infants [8], [9]. Total parenteral nutrition (TPN) is often an initial feeding strategy for preterm infants which may result in a distinct and probably delayed colonization compared to full term infants [10]. Extensive use of antibiotics further disturbs the natural diverse colonization in preterm infants, in combination with the specific properties of the immature intestinal tissue of preterm infants [9]. This phenomenon could, in part, explain the high incidence of NEC in the neonatal intensive care unit population [11].

Over the past 15 years, a number of studies have investigated the effects of probiotics (primarily *lactobacilli* and *bifidobacillia*) in preventing NEC. The most recent systematic review analyzed 20 clinical trials and concluded that probiotic supplementation was associated with a significantly decreased risk of NEC in preterm VLBW infants (RR = 0.33; P<.00001) [12]. The risk of death was also significantly reduced in the probiotic group (RR = 0.56; P<.0001). However, the optimal species of probiotic could not be ascertained, and research has yet to identify the major mechanisms by which probiotics prevent NEC. *Lactobacillus reuteri* DSM 17938 (LR17938) was derived from *L. reuteri* ATCC 55730 from a Peruvian mother’s breast milk by the removal of two plasmids harboring antibiotic resistance genes [13]. This strain inhibits pathogen growth and modulates the immune system. Immunomodulatory activity of LR17938 paradoxically includes a mild pro-inflammatory effect in monocytoid cells [14] and in intestinal epithelial cells *in vitro* [15]. Conversely, we have shown that feeding this strain to newborn rats produced a strong overall anti-inflammatory effect and reduced the incidence and severity of NEC *in vivo* [15].

Therefore, in this study, we initially characterized the phenotypic composition of T cell subsets in the ileum, mesenteric lymph nodes (MLN), spleen and thymus during the period of early neonatal development (DOL 0–4). We further determined the changes of T subsets in the ileum and MLN produced by NEC. Finally, we determined the effect of LR17938 supplementation on the various T cell subsets in the settings of dam-feeding, formula-feeding, and experimental NEC.

## Materials and Methods

### Probiotic *L. reuteri* DSM 17938 Preparation

Human breast milk-derived LR17938 was provided by Biogaia, Inc. (Stockholm, Sweden). LR17938 was anaerobic cultured in deMan-Rogosa-Sharpe (MRS; Difco, Detroit, MI) medium at 37°C for 24 h, then plated in MRS agar at specific serial dilutions and grown anaerobically at 37°C for 48–72 h. Quantitative analysis of bacteria in culture media was performed by comparing absorbance (at 600 nm) of cultures at known concentrations, using a standard curve of bacterial colony-forming units (CFU)/mL grown on MRS agar (using an Eppendorf Photometer, Eppendorf, Hamburg, Germany). Bacteria in the culture media were harvested by centrifugation at 1500 g for 15 min and were resuspended in formula before feeding.

### Animal Model and Experimental Design

Ethics Statement: Studies were approved by the Animal Welfare Committee of the University of Texas Health Science Center at Houston (# HSC-AWC-10-147).

All in vivo experiments were performed using newborn Sprague-Dawley rat pups (Harlan laboratories, Indianapolis, IN) weighing 5–6 g. To examine the effects of probiotic LR17938 on newborn rats under dam-fed or formula-fed conditions, two groups of newborn rats that stayed with their moms were fed with (Dam+LR17938) or without LR17938 (Dam) daily. The other two groups of newborn rats which were separated from their dams and housed in an incubator were fed with formula with or without 17938 from day of life 0 to 4 (n = 9 animals at each DOL/group, total n = 45 animals in each group). To examine the effects of probiotic LR17938 on newborn rats with NEC, rat pups were divided randomly into 3 groups. 1) Dam-fed normal controls: rat pups that were left with their mothers and were breast fed (Dam, n = 15). 2) Rat pups with NEC (formula-fed-hypoxic) (NEC, n = 22). One day old (DOL1) newborn rats were separated from the dam, housed in an incubator and starved for 12 h before the initiation of formula feeding on day 2 with 100–200 µl of formula, five times daily and to induce NEC were subjected to 10 min of hypoxia (5% oxygen, 95% nitrogen) three times daily in a Modular Incubator Chamber (Billups-Rothenberg, Del Mar, CA) for 2 days. The formula consisted of 15 g Similac 60/40 (Ross Pediatrics, Columbus, OH) in 75 ml of Esbilac canine milk replacement (Pet-Ag, Hampshire, IL) [16]. 3) Pups with NEC fed with LR 17938: the exact procedure was performed as NEC group except for formula containing LR17938 (10^6^ cfu/g.bw/day, NEC+17938, n = 22). Animals were monitored every three hours during 3-day period of study. No analgesia was offered to rats or mice in this experimental NEC model in previously published studies [17], [18]. However, if animals were in pain, demonstrating labored respirations, severe abdominal distension or gastrointestinal bleeding, we euthanized them at this point by peritoneal injection of pentobarbital (FatalPlus) at 1000 mg/kg. Otherwise, pups were euthanized on day 4 after live animal numbers were counted to collect tissues. The % of survival was calculated.

### Tissue Harvest and NEC Evaluation

Following incision of the abdomen, the gastrointestinal tract was carefully removed. The small intestine was evaluated visually for typical gross signs of NEC, such as intestinal distension, wall hemorrhage, or necrosis. The terminal 5 cm of small intestine (ileum) was excised. The terminal 1 cm of each sample was formalin fixed and processed by the Cellular and Molecular Morphology Core Lab (the Texas Medical Center Digestive Diseases Center, Houston, TX) and stained with hematoxylin and eosin (H&E) for histological evaluation. The remaining 4 cm of small intestine was used for isolation of lymphocytes. Histological NEC scores were defined as described previously [16], [19].

### Tissue Preparation for Flow Cytometry

Single cell suspensions from the spleen, thymus, and mesenteric lymph node (MLN) were obtained by gently fragmenting and filtering the tissues through 40 µm cell strainers (BD Bioscience) into MACS buffer consisting of phosphate buffered saline (PBS), 0.5% bovine serum albumin (BSA) (Hyclone Laboratories) and 2mM EDTA (Lonza). The red blood cells (RBCs) from the spleen were lysed using ACK lysing buffer (Quality Biological, Inc.). For the terminal ileum, tissue was incubated for 45 minutes at 37°C in RPMI-1640 (Sigma) complete medium containing collagenase V from clostridium histolyticum (Sigma, St. Louis, MO) at the concentrations of 0.01 mg/mL followed by vigorously vortexing for 1 min. Afterward, it was filtered through a 40 µm cell strainer.

### Flow Cytometry Analysis

Cells were surface stained using the following mouse anti-rat antibodies: CD3 (1F4), CD4 (OX-38), CD8a (OX-8) from Biolegend (San Diego, CA). Intracellular staining for Foxp3 was performed with fixation/permeabilization kit according to the manufacturer’s protocol (eBioscience, Dan Diego, CA) and detected with anti-Foxp3 (FJK-16 s, eBioscience). All samples were analyzed with BD FACSCalibur and processed with FlowJo (TreeStar, Inc. Ashland, OR).

### Immunohistochemistry (IHC)

CD3 immunohistochemistry staining was performed using a modified Milipore IHC Select kit protocol (Temecula, CA) according to manufacturer’s instruction. Briefly, tissue sections were baked at 60°C overnight and deparaffinized in xylene and hydrated in graded ethanol to water. Antigen retrieval was performed in boiling 10 mM sodium citrate buffer (pH 6.0) for 10 minutes in a microwave and cooled down for 30 minutes. Drops of 3% H_2_O_2_ were added to quench endogenous peroxides activity. Tissue sections were blocked with blocking buffer containing normal goat serum for 5 minutes, rinsed and incubated with 2 µg/ml of anti-CD3 antibody (Abcam, Cambridge, MA) as primary antibody for 1 hour. Afterward, goat anti-rabbit secondary antibody was used to detect rabbit anti-CD3. Streptavidin-HRP and Diaminobenzidine (DAB) was used to visualize region of tissue with anti-CD3. Hematoxylin was used to counter stain cells. Digitized images of stained tissues were taken using Olympus BX51 (Pennsylvania, USA) microscope at 200 × magnification.

### Statistics

Experimental results are expressed as means ± SE. Statistical analysis was performed using two-way ANOVA, one-way ANOVA and correlation (Graph Pad Prism 4.0; GraphPad Software, San Diego, CA). Two-way ANOVA analysis was used when comparing % of each different T subtypes at DOL1, DOL2, DOL3 and DOL4 compared to DOL0, respectively, and comparing % of each different T subtypes between two groups such as dam-fed vs. dam+17938 or formula vs. formula+17938. One-way ANOVA was used when compared the groups at the same DOL. Dunnett’s and Tukey’s multiple-comparison tests were used for comparison of multiple groups with a control group. Chi-square test was used for comparison of survival (%) between groups of dam vs. NEC, or NEC vs. NEC+17938. A p value <0.05 was considered statistically significant. The correlation of % of CD4^+^Foxp3^+^ with % of CD4^+^CD8^+^Foxp3^+^, and the correlation of % of CD4^+^Foxp3^+^ with NEC scores in the intestines of rats with NEC+17938 were performed by using linear regression analysis.

## Results

### Phenotypic Composition of T cell Subsets in the Ileum, Mesenteric Lymph Nodes, Spleen, and Thymus of Normal Rats during Early Postnatal Life

The fully-mature intestinal mucosa contains a large number of lymphocytes in the epithelium and lamina propria in humans and rodents. Most of these lymphocytes are T cells. Any organism that is able to traverse the intestinal epithelium will encounter these lymphocytes. However, little information is available on the composition of T cell subsets in neonatal intestine. To understand the characteristics of T cells in the neonatal intestine and how probiotic LR17938 modulates the Foxp3^+^ Tregs in the intestines after oral administration to newborn rats, we first examined the phenotypic composition of T cell subsets in the distal 4-cm of terminal ileum from day of life (DOL) 0 to DOL 4 during the course of NEC in a neonatal rat model. These T cell subsets were characterized by labeling markers which included CD3 (T cells), CD4 (T helpers), CD8α (cytotoxic T cells) and intracellular Foxp3 (regulatory T cells). The identification of T cell subsets by flow cytometry analysis is shown in Figure S1. For all organs we studied (ileum, MLN, spleen and thymus), we gated CD3^+^ T cells in the population of lymphocytes, and further defined the percentage of single CD4^+^, single CD8^+^ and double CD4^+^CD8^+^ T cells. Frequency of Foxp3^+^ T cells were characterized by single CD4^+^Foxp3^+^ Tregs (solid red circle) or double CD4^+^CD8^+^Foxp3^+^ T cells (dashed red circle) gated by single CD4^+^ T cells and double CD4^+^CD8+ T cells, respectively.

**Figure 1:**
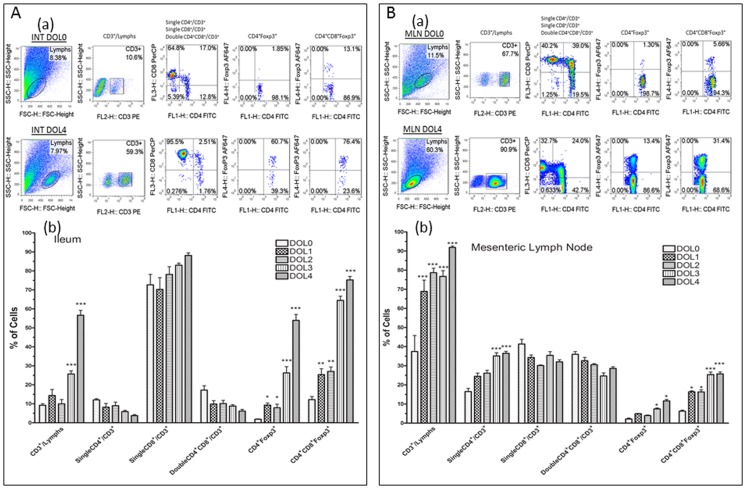
Phenotypic composition of T cell subsets in the ileum and mesenteric lymph nodes (MLN) of normal rats during day of life (DOL) 0 to DOL 4. The frequencies of T cell subsets including CD3^+^, CD4^+^, CD8^+^, CD4^+^CD8^+^, CD4^+^Foxp3^+^, and double positive CD4^+^CD8^+^Foxp3^+^ cells, and the comparisons between DOL 0 and DOL 4 were shown by representative flow cytometry plots in ileum (1A–a) and mesenteric lymph node (1B–a) of newborn rats. The bars reflect the percentage of cells, shown as means ± SE, N = 9 pups at each DOL. Comparisons were made between on DOLs 1, 2, 3, or 4 compared with DOL0 in the ileum (1A–b) or MLN (1B–b) of newborn rats, respectively. *p<0.05, **p<0.01, and ***p<0.001.

Results indicated that during days of life 0 to 4, in the ileum (Figure 1A), the percentage of CD3^+^ T cells among lymphocytes in the ileum was 9.2±0.8% on DOL 0 but increased to 25.7±1.6% on DOL 3 and 56.6±2.6% on DOL 4 (p<0.001). The majority of CD3^+^ cells showed the CD8α phenotype (>80%) on DOL 4. The population of CD4^+^ or double positive CD4^+^CD8^+^ T cells among CD3^+^ T cells represented about 10% of the T cells during DOL 0 to 4.

We found that positive staining of intracellular Foxp3 was not only present on single CD4^+^ cells but also on the double CD4^+^CD8^+^ population. The percentage of Foxp3^+^ cells in single CD4^+^ cells was miniscule at DOL 0 (1.8±0.2%) but significantly increased after pups were fed by their dams for only one day (9.3±1.2%, p<0.05). This population of regulatory cells dramatically increased to 52.6±3.2 on DOL 4 (p<0.001) (Figure 1A). Surprisingly, the percentage of Foxp3^+^ staining on double positive CD4^+^CD8^+^ cells was much higher (12.2±1.6%) on DOL 0. Similar to ontogenic chances in CD4^+^/Foxp3^+^ intestinal lymphocytes, CD4^+^CD8^+^ Foxp3^+^ cells increased by 2-fold on DOL 1 (25.4±3.1%) and by 4-fold on DOL 4 (75.2±1.8%) compared to DOL 0 (p<0.001) (Figure 1A). It is noteworthy that the absolute number of Foxp3^+^ cells from 4-cm terminal ileum is very low for 0 to 4-day old neonatal rats because CD3^+^ cells represent <10% of total cells.

In contrast, in mesenteric lymph nodes (Figure 1B), the percentage of CD3^+^ cells among lymphocytes was high and significantly increased on DOL 1 to DOL 4 compared to DOL 0, respectively, all p<0.001. Almost all the lymphocytes in the mesenteric lymph nodes on DOL 4 were CD3^+^ T cells (91.9±0.8%). The % of CD4^+^ cells in CD3^+^ T cells was also significantly increased in the mesenteric lymph nodes of rats on DOL 4 compared to DOL 0 (p<0.001). The percentages of single CD4^+^, single CD8^+^ and double CD4^+^CD8^+^ cells among CD3^+^ T cells averaged about 30% on DOL 4. Foxp3^+^ staining was present on single CD4^+^ and double CD4^+^CD8^+^ cells. These Foxp3^+^ cell populations also increased in a day of life-dependent manner (Figure 1B). The percentages of CD4^+^Foxp3^+^ and CD4^+^CD8^+^Foxp3^+^ T cells were significantly increased on DOL 4 compared to DOL 0, respectively (p<0.05 for CD4^+^Foxp3^+^ cells and p<0.001 for CD4^+^CD8^+^Foxp3^+^ cells).

These findings in the MLNs were mirrored by T cell subsets isolated from whole spleen of neonatal rats (Figure S2). Foxp3^+^ staining was present on single CD4^+^ and double CD4^+^CD8^+^ cells, and the Foxp3^+^ cell populations increased in a day of life-dependent manner.

In the thymus, approximately 99% of lymphocytes were CD3^+^ T cells, mostly (∼80%) bearing the double CD4^+^CD8^+^ phenotype. Foxp3^+^ T cells represented less than 5% of CD4^+^ cells in the neonatal thymus (Figure S3). There was no significant change in the number of Foxp3 cells in the thymus during the first 4 days of life.

### Effect of Feeding Method on Foxp3^+^T cells in the Ileum and Mesenteric Lymph Nodes after Birth

**Figure 2:**
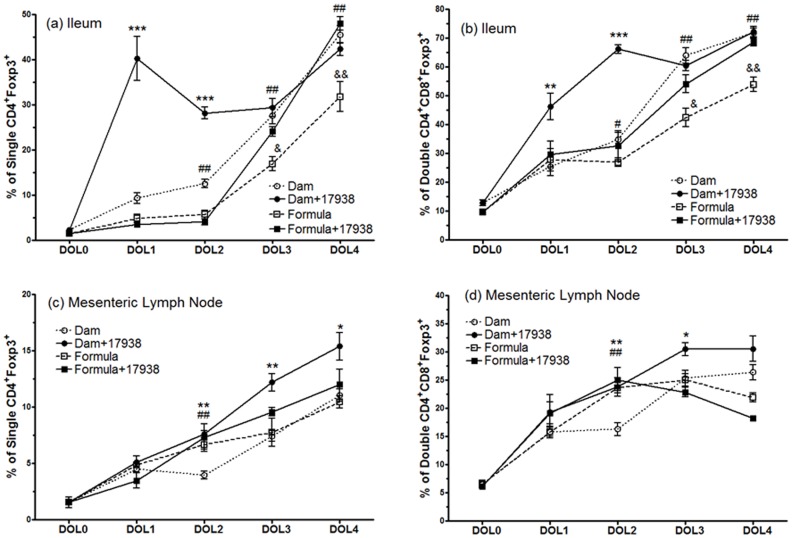
Effect of breast milk or formula feeding with or without *Lactobacillus reuteri* DSM 17938 on the frequency of Foxp3^+^ T cells in the ileum and mesenteric lymph node (MLN) of rats during DOL 0 and DOL 4. Newborn rats were fed immediately after birth for 4 days. (a) and (c): Changes of single CD4^+^Foxp3^+^ Tregs (%) in the ileum (a) and MLN (c); (b) and (d): changes of double CD4^+^CD8^+^Foxp3^+^ T cells in the ileum (b) and MLN (d). The percentage (%) of cells is represented as the mean ± SE of N = 9 observations (pups) in each DOL of each feeding group (total N = 45 for each feeding group).* Dam-fed +17938 vs. Dam-fed, **p<0.01, ***p<0.001. # Formula vs. Dam-fed, # p<0.05, ## p<0.01. &: Formula-fed +17938 vs. Formula-fed, & p<0.05, && p<0.01.

We proposed that LR17938 might facilitate the generation of Foxp3^+^ Tregs in the terminal ileum of neonatal rats. We determined the changes in percentage of Foxp3^+^ T cells from DOL 0 to DOL 4 among the feeding groups (dam, dam+LR17938, formula, and formula+LR17938) (Figure 2a and 2b). We found that the percentages of CD4^+^Foxp3^+^ Tregs and double positive CD4^+^CD8^+^Foxp3^+^ T cells significantly increased in the ileum of rats as early as DOL 1 and DOL 2 after orally feeding LR17938 immediately after birth, compared to numbers in the rats without probiotic feeding (CD4^+^Foxp3^+^ Tregs: 40.2±0.2% in Dam+17938-fed vs. 9.3±1.2% in Dam-fed at DOL 1, p<0.001, and 28.1±1.3% in Dam+17938-fed vs. 12.5±0.9% in Dam-fed at DOL 2, p<0.001, Figure 2a. In addition, CD4^+^CD8^+^Foxp3^+^ T cells in the ileum increased: 46.2±1.7% in Dam+17938-fed vs. 25.4±3.1% in Dam-fed at DOL 1, p<0.01, and 66.1±1.5% in Dam+17938-fed vs. 34.8±2.4% in Dam-fed at DOL 2, p<0.001, Figure 2b). However, no significant differences of Foxp3^+^ T cells were observed at DOL 3 and DOL 4 compared to dam-fed only (Figure 2a and Figure 2b). This finding led us to investigate the change of T cell subsets under formula-feeding conditions.

In formula-fed rat pups, we did not observe a change of CD4^+^Foxp3^+^ T cells or double positive CD4^+^CD8^+^Foxp3^+^Tregs in the terminal ileum of neonatal rats on DOL 1 and DOL 2 after orally feeding 17938. However, feeding LR17938 produced an increase in the percentages of both single positive CD4^+^Foxp3^+^ (Figure 2a) and double positive CD4^+^CD8^+^Foxp3^+^ (Figure 2b) Tregs in the ileum on DOL 3 (p<0.05) and DOL 4 (p<0.01), compared to Treg percentages in rats fed with formula without probiotic. Treg percentages in formula-fed rats were significantly lower than in dam-fed rats on DOL 2 to DOL 4 (p<0.01).

Mesenteric lymph nodes are the repository of lymphocytes and other immune cells that communicate between central immune organs such as the spleen and thymus and the intestinal lumen. Lymphocytes including Tregs may be recruited from the MLN to the intestinal epithelium and lamina propria in various diseases. We have shown that CD3^+^ T lymphocytes are the dominant cells in the MLN of neonatal rats, especially on DOL 4 (∼90% of lymphocytes) (Figure 1b). We found that LR17938 supplementation produced an increase in % of single CD4^+^Foxp3^+^ Tregs in the MLN of neonatal rats on DOL 2 (7.6±0.3 vs. 3.9±0.3, p<0.01), DOL 3 (12.2±0.8 vs. 7.4±0.5, p<0.01) and DOL 4 (15.4±1.2 vs. 11.0±0.5, p<0.05) compared to Tregs in the MLNs of neonatal rats suckling mother’s breast milk (Figure 2c). Similarly, feeding with LR 17938 to the pups with dam also increased double CD4^+^CD8^+^Foxp3^+^ T cells on DOL 2 and 3 compared to dam-fed without probiotic (p<0.01) (Figure 2d). However, we didn’t observe any change of MLN Treg percentages in LR17938-supplemented formula-fed rats.

### Impact of NEC on T cell Subsets in the Ileum and Effects of *L. reuteri* DSM17938

**Figure 3:**
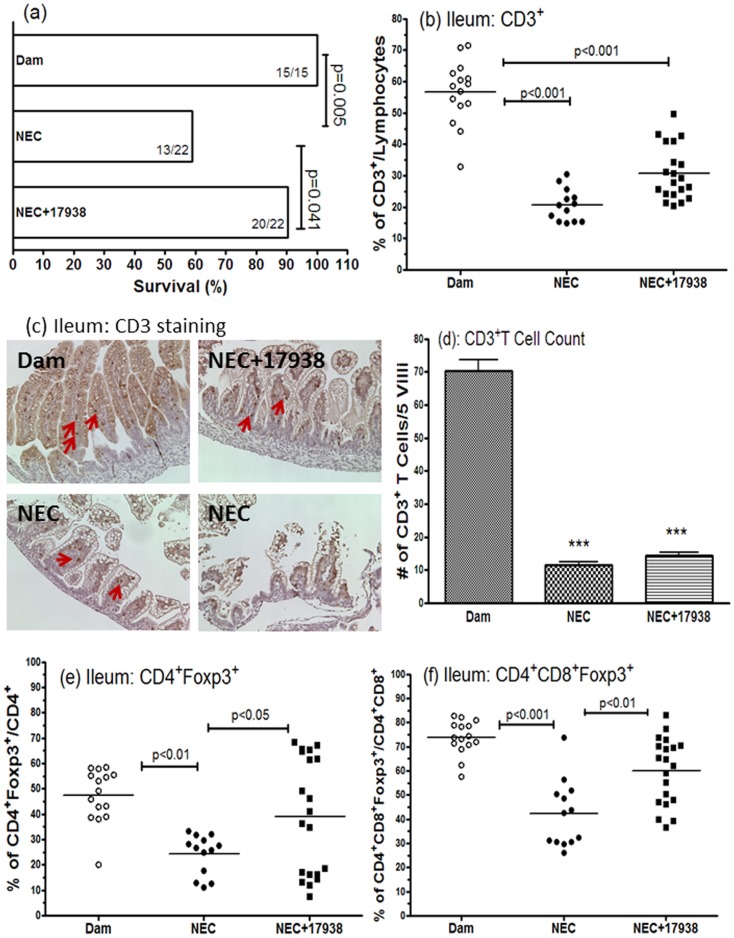
Effect of *Lactobacillus reuteri* DSM 17938 on survival, CD3^+^ T cells and Foxp3^+^ T cells in the ileum of rats with NEC. (a) Survival (%) of different groups of rat pups (dam-fed, NEC, and NEC+17938). (b) Changes of % CD3^+^ T cells of lymphocytes in different groups. (c) Immunohistochemical staining of CD3^+^ cells in rat pup ileum, 200 × magnification. (d) Number of CD3^+^ T cells per 5 villi in IHC. (e) Percentage of CD4^+^Foxp3^+^Tregs in each group on day 4 of NEC. (f) Percentage of double positive CD4^+^CD8^+^Foxp3^+^ T cells. The numbers represent percent of T cells (means ± SE). Each dot represents one animal in each group in the figures b, e, and f. Dam-fed: N = 15, NEC: N = 13, NEC+17938: N = 20.

We have previously reported that LR17938 increased the survival of rat pups with NEC [19]. In the current study, we also measured survival because only live animals could be used to collect immune cells from the tissues. In the dam-fed group, none of the pups died (survival rate = 100% (15/15)), whereas in the NEC group the survival rate was 59% (13/22) (p = 0.005). Survival was 90% (20/22) in NEC+17938 group (p = 0.041, compared to NEC without probiotic treatment), indicating LR17938 increased the survival rate of rats with NEC (Figure 3a).

It is unknown if insufficient numbers of Tregs contributes to the pathogenesis of NEC and if the effect of LR 17938 on NEC is associated with alterations in T cell subsets in the intestine. In this study, we characterized the T cell subsets of the ileum in rats with NEC compared to dam-fed normal controls, as well as in experimental NEC under the condition of LR17938 feeding. Results indicated that the percentage of CD3^+^ T cells in the terminal ileum of neonatal rats with NEC significantly decreased compared to percentage in ilea of dam-fed control rats (Figure 3b) (p<0.001).

Immunohistochemical staining of CD3^+^ T cells in formalin-fixed ileal sections of rats allowed us to visualize CD3^+^ T cell staining in NEC compared to dam-fed control along with histological injury in NEC (Figure 3c). These T cells were largely localized to the villus cores and lamina propria. The numbers of CD3^+^ T cells per 5 villi counted in the images of immunohistochemical staining were significantly decreased in NEC (11.6±0.9 in NEC vs. 70.4±3.2 in dam-fed rats, p<0.001) (Figure 3d).

In addition, Foxp3^+^ Treg cells including single positive CD4^+^Foxp3^+^ T cells (Figure 3e, p<0.01), and double positive CD4^+^CD8^+^Foxp3^+^ T cells (Figure3f, p<0.001) significantly decreased in the ileum of rats with NEC, compared to normal dam-fed rats. Feeding LR17938 to NEC rats did not produce an increase in the percentage of CD3^+^ T cells (Figure 3b) and numbers of CD3^+^ T cells per villus (Figures 3c and 3d). However, in response to LR17938, the level of CD4^+^Foxp3^+^ Tregs and double positive CD4^+^CD8^+^Foxp3^+^ Tregs returned to normal in the ileum of rats with NEC (compared to untreated NEC animals, p<0.05 and p<0.01, respectively) (Figure 3e and 3f). There were no changes of % of CD4^+^/CD3^+^, CD8^+^/CD3^+^, or double positive CD4^+^CD8^+^/CD3^+^ T cells during NEC, with no influence of LR17938 feeding (data not shown).

We noticed the heterogeneity in the distribution of percentage of CD4^+^Foxp3^+^ and CD4^+^CD8^+^Foxp3^+^ T cells in the ileum within the group of NEC+LR17938. We observed that newborn rats with low percentages of single and double positive Tregs in Figure 3e and Figure 3f were the same individuals. The correlation between percentage of intestinal CD4^+^Foxp3^+^ T cells and percentage of intestinal CD4^+^CD8^+^Foxp3^+^ T cells was significant (p = 0.0275 and r = 0.4920).

In addition, the animals with a high percentage of Foxp3^+^ cells had lower NEC scores than animals with a low percentages of Foxp3^+^ cells within the group of NEC+LR17938: there was a significant correlation between % CD4^+^Foxp3^+^ T cells and NEC score (p = 0.0219 and r = − 0.5089).

### T cell Subsets in the Mesenteric Lymph Nodes of Neonatal Rats with NEC and Effects of *L. reuteri* DSM17938 Feeding

**Figure 4:**
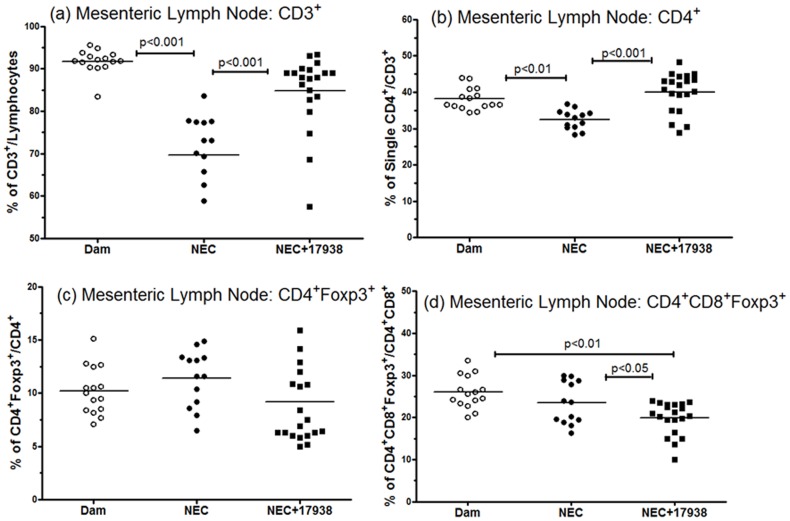
Effect of *Lactobacillus reuteri*DSM 17938 on CD3^+^, CD4^+^ and Foxp3^+^ T cells in the mesenteric lymph nodes of rats with NEC. (a): changes of CD3^+^ T cells of lymphocytes, (b) % of CD4^+^ T cells/CD3^+^ T cells, (c) % of CD4^+^Foxp3^+^, and (d) double positive CD4^+^CD8^+^Foxp3^+^ cells in the mesenteric lymph nodes in different groups. The numbers represent percent of T cells (mean ± SE). Each dot represents one animal in each group in the figures. Dam-fed: N = 15, NEC: N = 13, NEC+17938: N = 20.

Changes of T cell subsets in the mesenteric lymph nodes of neonatal rats with NEC and the effect of LR17938were identified in this study. Results showed that the percentage of CD3^+^ T cells (compared to total lymphocytes) in NEC was lower than in normal rat pups (74.7±2.9 in NEC vs. 91.9±0.8 in dam-fed rats, p<0.001) (Figure 4a). Similarly, the % of single CD4^+^ T cells compared to total CD3^+^ T cells during experimental NEC was lower (32.7±0.6 in NEC vs. 37.9±0.8 in dam-fed rats, p<0.01)(Figure 4b) in the mesenteric lymph nodes. The ratio could be restored to normal by feeding LR17938 to the rat pups with NEC (% CD3^+^: 84±2.4, p<0.05 vs. NEC, and % CD4^+^: 40±1.2, p<0.01 vs. NEC) (Figure 4a and Figure 4b). The percentages of either single positive CD4^+^Foxp3^+^ Tregs or double positive CD4^+^CD8^+^Foxp3^+^ T cells in MLN’s did not change in animals with NEC compared to dam-fed controls. However, LR17938 significantly decreased the percentage of double CD4^+^CD8^+^Foxp3^+^ T cells in the MLN compared to NEC rats without probiotic (20.6±0.7 in NEC+LR17938-fed vs. 24.5±1.8 in NEC, p<0.05) (Figure 4d). This finding in combination with the increase in doubly-positive Tregs in the intestinal tissue might point toward migration of doubly positive Tregs from the MLN to the site of active inflammation (the gut) during NEC promoted by LR17938.

There were no LR-induced or formula-induced changes in T cell subsets observed in the spleen or thymus (data not shown).

## Discussion

Beginning at birth and throughout life, the mucosal surface of the small intestine is constantly exposed to a large variety of foreign antigenic materials, such as dietary macromolecules, food proteins, and resident microbiota, with a resulting state of constant antigenic stimulation. Information exchange between immune cells, enterocytes, and microbes plays an important role in the maturation of the immune system [20]. The intestinal mucosa contains a large number of lymphocytes (most of them T cells) in the epithelium in fully mature humans and rodents. Recent studies indicate that Foxp3^+^ regulatory T cells (Tregs) are responsible for maintaining gut homeostasis, controlling inflammation and inducing tolerance [5]. However, little information is available on how these lymphocytes develop in the early stages of life.

We have recently shown that LR17938 reduces the incidence and severity of experimental necrotizing enterocolitis via modulation of TLR4 and NF-κB signaling in the intestine [19]. We hypothesized that the susceptibility to NEC is due to a deficiency in Tregs, resulting in a dysregulated immune response to foreign antigens in the developing intestine, a defect which could be modulated by administration of 17938. In the current study, we characterized the T lymphocyte phenotypes including single CD4^+^, single CD8α^+^, single CD4^+^Foxp3^+^ and double CD4^+^CD8^+^Foxp3^+^ cells in the ileum, mesenteric lymph node, spleen and thymus of neonatal rats during the period of day of life 0 to 4.

We herein have identified five important findings related to the development of Tregs: (1) During normal development, there is a rapid increase in the frequency of intestinal Foxp3^+^Tregs among single CD4^+^ and double CD4^+^CD8^+^T cells early in life. (2) The increase in intestinal Tregs can be accelerated by LR17938 administration to rat pups fed breast milk or formula (more rapidly in the former group than in the latter). (3) During NEC, significant intestinal inflammation is associated with a decrease in the frequency of intestinal Foxp3^+^Tregs in the single positive CD4^+^ and double positive CD4^+^CD8^+^ T cell populations. (4) We have shown that LR17938 treatment prevents NEC while increasing the frequency of intestinal CD4^+^Foxp3^+^Tregs and CD4^+^ CD8^+^Foxp3^+^ T cells. (5) During NEC, in the mesenteric lymph nodes, the frequency of CD3^+^ T cells in the total lymphocyte population and the percentage of single CD4^+^ T cells among the population of T cells were decreased, however, the number of these cells could be restored by feeding 17938. Feeding LR17938 significantly decreased the percentage of double CD4^+^CD8^+^Foxp3^+^ T cells in MLN compared to dam-fed rats or rats with NEC that did not receive probiotic feeding, suggesting that probiotics might promote the migration of double positive Tregs from the MLN to the site of active inflammation (the gut).

It is noteworthy that even though we detected an approximately 4-fold increase in the percentage (%) of CD3^+^ T cells among lymphocytes in the ileum on DOL 4 compared to DOL 0, in fact, the absolute total number of CD3^+^ T cells in the distal ileum was very low at this early stage. As an example, among 100,000 total cells only 1,000 cells were CD3^+^ T cells on DOL 0, the number increasing to 5,000 cells on DOL 4. A striking finding in newborn rats was that the majority of CD3^+^ T cells displayed the single CD8α phenotype (>80%), which has traditionally been viewed as cytotoxic T cells. Single CD4^+^ or double CD4^+^CD8^+^ populations among CD3^+^ T cells represented less than 10% of these T cells from DOL 0 to 4. This breakdown is different from that seen in adult humans or rats. Our results in adult rats and other studies in humans and rats [21], [22] show a predominance of single positive CD4^+^ lymphocytes in the intestine, followed by B cells. Only about 20% of all lymphocytes are CD8^+^ T cells in mature humans and rats (data not shown).

The function of the abundant single CD8^+^ T cells in very early neonatal life is not completely understood. This subset may be the first intestinal lymphocyte to deal with food antigens, commensal organisms, and pathogens. Previous studies indicated a role for CD8^+^ T cells in the in *vivo* suppression of autoimmunity [23] and a number of CD8^+^ T cell clones demonstrated inhibitory activity [24]–[27].

Regulatory T cells are critical for intestinal immune homeostasis, and premature human infants are at increased risk of gut inflammation (milk protein allergy, NEC) appear to have low numbers of these cells. These infants may have a relative lack of Tregs in the intestinal lamina propria. Prior studies examining the ontogeny of lymphocytes in human fetal gastrointestinal tissue showed that T cells are present as early as 14 weeks gestation; they are seen well developed Peyer’s patches with germinal centers and T cell rich zones as early as week 19 [28]. Intraepithelial and lamina propria lymphocytes are also present in fetal intestinal tissues [29]. In the human intestinal tract, Tregs are present immediately after birth in babies, as shown by immunohistochemical staining [30].

Our model of NEC is imperfect and involves the stresses of maternal separation, under nutrition, and hypoxia, but it does produce a reproducible histological lesion that is not dissimilar to that of human NEC [16], [18]. This model and similar models have shown mechanisms by which activation of the classical toll-like-receptor-4 signaling pathway leads to production of many proinflammatory cytokines, observations that also apply to human babies [16]. Little is known about the ontogeny of Tregs in the intestine and mesenteric lymph nodes of newborn rats during the first days of life. Our current studies demonstrate that Foxp3^+^ T cells increase from very low levels during early ontogenesis in the intestine, MLN and spleen, while remaining very low in the thymus of neonatal rats. When neonatal rats suckled breast milk, Treg development was rapid and was further stimulated by LR17938.

LR17938 combined with suckling to increase the percentages of single CD4^+^Foxp3^+^Tregs within 24 h (on DOL 1). This phenomenon was not observed in the formula-fed animals, which were separated from their mothers immediately after birth. Feeding 17938 to formula-fed animals at DOL 0 did increase the percentage of Foxp3^+^Tregs, but the effect was delayed until DOL 3. These results imply that some factor in breast milk may enhance the immunomodulatory effects of LR17938. It has been known that breast milk contains lactose, whey, casein and different oligosaccharides that are believed to favor the growth of certain bacterial species [31]. Breast milk also contains a variety of bioactive components such as IgA, lactoferrin, β-lactoglobulin and α-lactabumin that directly or indirectly modulate the immune system [32]–[34]. Human milk contains up to 10^9^ CFU per ml of live bacteria with a dominance of *Bifidobacteria* [35]. Maternal milk is the major source of epidermal growth factor (EGF) for neonates, which plays an important protective role against NEC development [36]. The protective effect of maternal milk is associated with increased production of mucosal IL-10 in the site of injury, indicates an immunomodulatory activity [37]. The interaction of these factors with intraluminal LR17938 during immunomodulation should be further explored.

The immune system of the neonatal intestine under certain circumstances mounts an exaggerated, uncontrolled response to the colonizing bacteria [38]. A recent study analyzed lamina propria mononuclear cell populations from surgically resected ileum from patients with NEC and gestational age-matched non-NEC surgical controls. The investigators found that in NEC tissue there was a reduction in Tregs which correlated with a mucosal cytokine expression profile that would be expected to inhibit the induction of Tregs [30]. Our current studies also demonstrated a decreased frequency of Foxp3^+^Tregs (single CD4^+^ and double CD4^+^CD8^+^ T cells) in the ileum during NEC. Tregs constitute a heterogeneous population has been bolstered by the identification of a population of CD8^+^ Foxp3^+^ T cells in autoimmune disorders and after allergen exposure [39], [40]. The potential importance of this cell population is highlighted by the recent demonstration that in humans with stem cell transplants for autoimmune disorders, the numbers of CD8^+^ Tregs correlated inversely with the level of ongoing inflammation [41]. In these studies, CD8^+^ cells were positively selected and isolated by anti-CD8 microbeads, then stained with anti-CD25 or Foxp3. The functions of CD8^+^CD25^+^ or CD8^+^Foxp3^+^ T cells were further studied. The authors did not pursue the examination of the expression of CD4 on these cells probably because the % of double CD4^+^CD8^+^ T cells is low in both adult human and mouse.

We observed that CD8^+^Foxp3^+^ cells constituted only 1–2% of T cells in the intestine, and only 3–5% of T cells in MLN of neonatal rats. The probiotic LR 17938 could not change the percentage of this subtype (data not shown). The decreased frequency of CD4^+^Foxp3^+^ and CD4^+^CD8^+^Foxp3^+^ Tregs in the intestine of neonatal rats with NEC combined with the evidence of increased local proinflammatory cytokines in this model [16], gives a clearer picture in which severe inflammation persists without downregulation and eventually leads to severe outcomes including surgery in many cases. Recent studies showed that intermittent depletion of Foxp3^+^Treg aggravates intestinal inflammatory responses [42], while adoptive transfer of Tregs prevents the onset of NEC in our lab (manuscript in preparation).

Despite limited evidence for a role of specific bacteria in the development of NEC [1], numerous clinical and laboratory trials [12] have been undertaken to modify intestinal colonization with probiotic bacteria to decrease the risk of NEC. When LR17938 was administered to neonatal rats with NEC, the frequency of single CD4^+^ Foxp3^+^ and double CD4^+^CD8^+^Foxp3^+^ Tregs increased to the same levels of dam-fed controls in the intestine. Simultaneously, the percentage of double positive CD4^+^CD8^+^Foxp3^+^ T cells decreased in the mesenteric lymph node, which suggests migration of the double positive Tregs from the mesenteric lymph node to the site of active inflammation promoted by LR17938. Because mortality was already reduced by DOL 4, we may infer that major change in percentage of Tregs induced by LR17938 may occur later than the changes in TLR signaling pathways that are a key component of its protective effect. Nevertheless, this effect on Tregs may contribute to its efficacy.

Different strains of probiotic bacteria are known to have different immunomodulatory effects. We have tested *L. reuteri* strain ATCC4659 in previous studies [15], [19]. We found that its effect of immune cell subtypes in the intestine and mesenteric lymph nodes of neonatal rats was slightly different than that of LR17938. ATCC 4659 had no effect on Tregs but was associated with a decrease in CD8^+^ T cells in the intestine of newborn rats (unpublished data). We also examined the effect of *Lactobacillus acidophilus* DDS (La DDS) on T cell subsets in the intestine and mesenteric lymph nodes in newborn rats. We did not observe a change in the percentage of Tregs when giving La DDS during the first 5 day of life of rats. La DDS has been shown to be lacking the properties of adherence to epithelial cells, induction of mucin expression by intestinal epithelial cells, and inhibition of *enteropathogenic* E. coli adherence to epithelial cells [43]. We also reported previously that La DDS has no effects on LPS-induced NFκB activation in the intestines of newborn rats in an ex vivo experiment [19].

LR17938 prevents NEC and therefore it is not surprising that LR prevents the associated decrease in Tregs in the intestine in NEC. LR17938 either restores CD3^+^ and CD4^+^ T cells in the MLN during the course of NEC or (as an alternative explanation) or prevents NEC and NEC associated changes in the lymphocyte composition of the MLN.

The mechanisms underlying NEC and the evolving picture of cross-talk between probiotics and intestinal epithelial cells, epithelial cells and immune cells, as well as how probiotics modify the function of immune cells in neonatal intestinal mucosa need to be further explored.

## Supporting Information

Figure S1 **Definition of T cell subsets by flow cytometry analysis.** Single cell suspensions from the ileum, MLN, spleen or Thymus were labeled for CD3, CD4, CD8α surface markers and intracellular Foxp3. Lymphocyte population was gated initially, followed by gating CD3^+^ T cell population in lymphocytes. Subsequently, the percentages of CD4^+^, CD8^+^, double positive CD4^+^CD8^+^ cells among CD3^+^ T cells, CD4^+^Foxp3^+^/CD4^+^ Tregs (solid red circle), and double positive CD4^+^CD8^+^Foxp3^+^/CD4^+^CD8^+^ T cells (dashed red circle) were defined. The data shown were analyzed from rat pups on day of life (DOL) 3.
(TIF)

Figure S2 **Phenotypic composition of T cell subsets in the spleen of normal rats during DOL 0 to DOL 4.** The bars reflect the percentage of cells, shown as means ± SE, N = 9 pups at each DOL. Comparisons were made between DOLs 1, 2, 3, or 4 compared with DOL 0. *p<0.05, **p<0.01, and ***p<0.001.
(TIF)

Figure S3 **Phenotypic composition of T cell subsets in the thymus of normal rats during DOL 0 to DOL 4.** The bars reflect the percentage of cells, shown as means ± SE, N = 9 pups at each DOL. There were no significant differences between DOLs 1, 2, 3, or 4 compared with DOL 0. Thymocytes are >80% CD3^+^CD4^+^CD8^+^ T cells.
(TIF)
