# Supplementary figures and images for: Lactobacillus reuteri DSM 17938 Changes the Frequency of Foxp3+ Regulatory T Cells in the Intestine and Mesenteric Lymph Node in Experimental Necrotizing Enterocolitis
Source: PLoS One. 2013 Feb 20;8(2):e56547. doi: 10.1371/journal.pone.0056547 (PMC3577854; doi:10.1371/journal.pone.0056547)

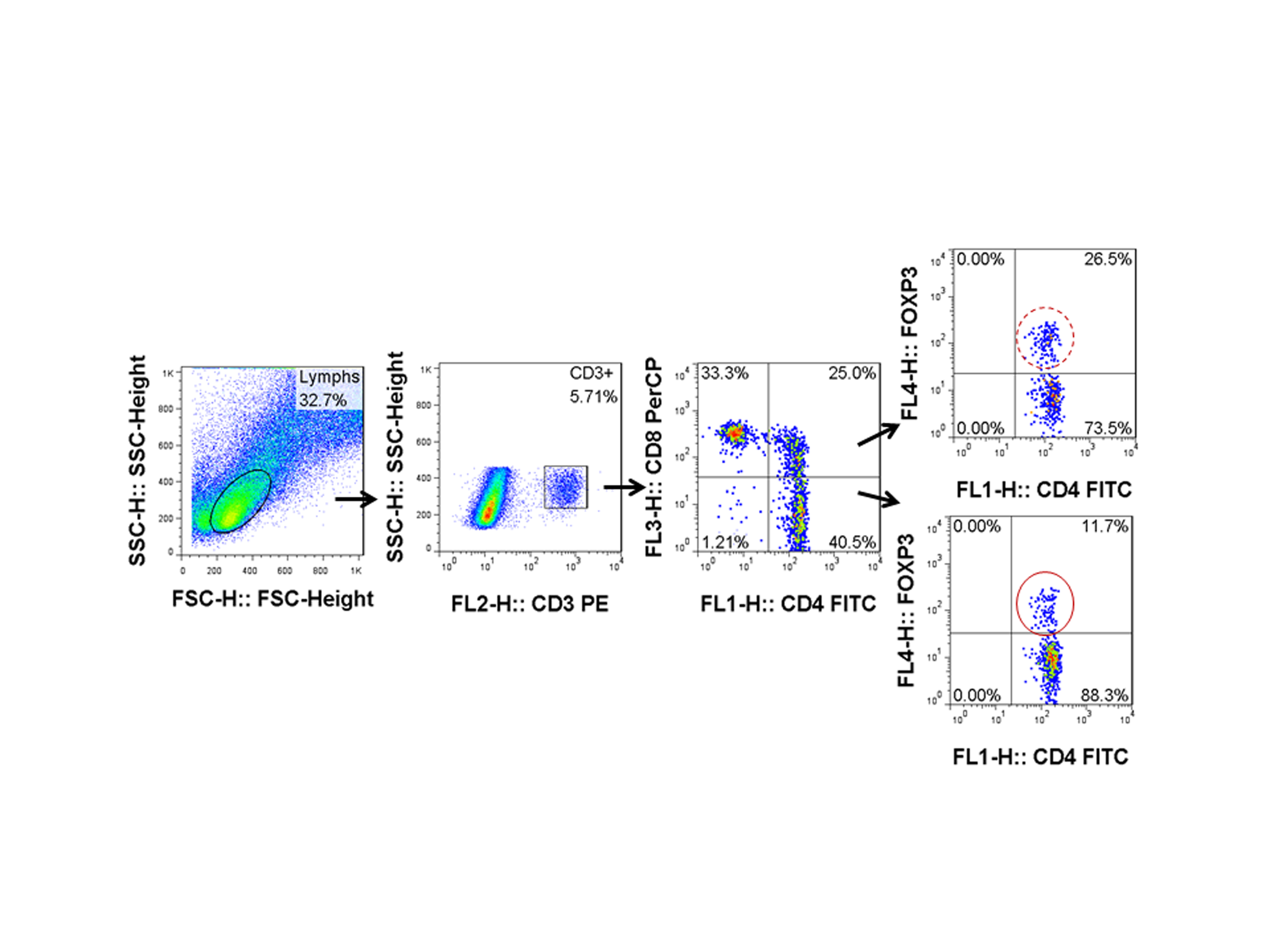

Supplement: Figure S1 — Definition of T cell subsets by flow cytometry analysis. Single cell suspensions from the ileum, MLN, spleen or Thymus were labeled for CD3, CD4, CD8α surface markers and intracellular Foxp3. Lymphocyte population was gated initially, followed by gating CD3+ T cell population in lymphocytes. Subsequently, the percentages of CD4+, CD8+, double positive CD4+CD8+ cells among CD3+ T cells, CD4+Foxp3+/CD4+ Tregs (solid red circle), and double positive CD4+CD8+Foxp3+/CD4+CD8+ T cells (dashed red circle) were defined. The data shown were analyzed from rat pups on day of life (DOL) 3. (TIF) [file pone.0056547.s001.tif]

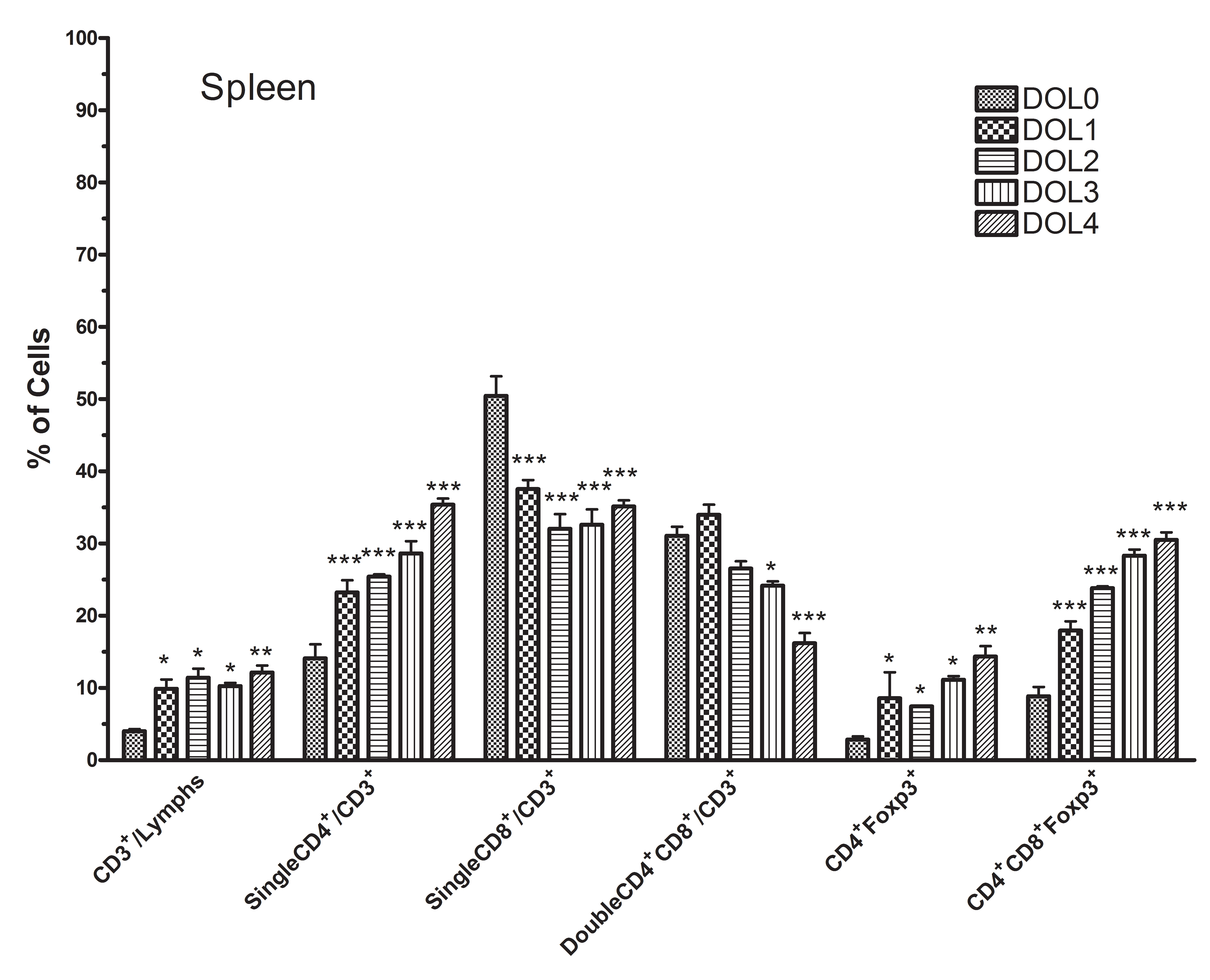

Supplement: Figure S2 — Phenotypic composition of T cell subsets in the spleen of normal rats during DOL 0 to DOL 4. The bars reflect the percentage of cells, shown as means ± SE, N = 9 pups at each DOL. Comparisons were made between DOLs 1, 2, 3, or 4 compared with DOL 0. *p<0.05, **p<0.01, and ***p<0.001. (TIF) [file pone.0056547.s002.tif]

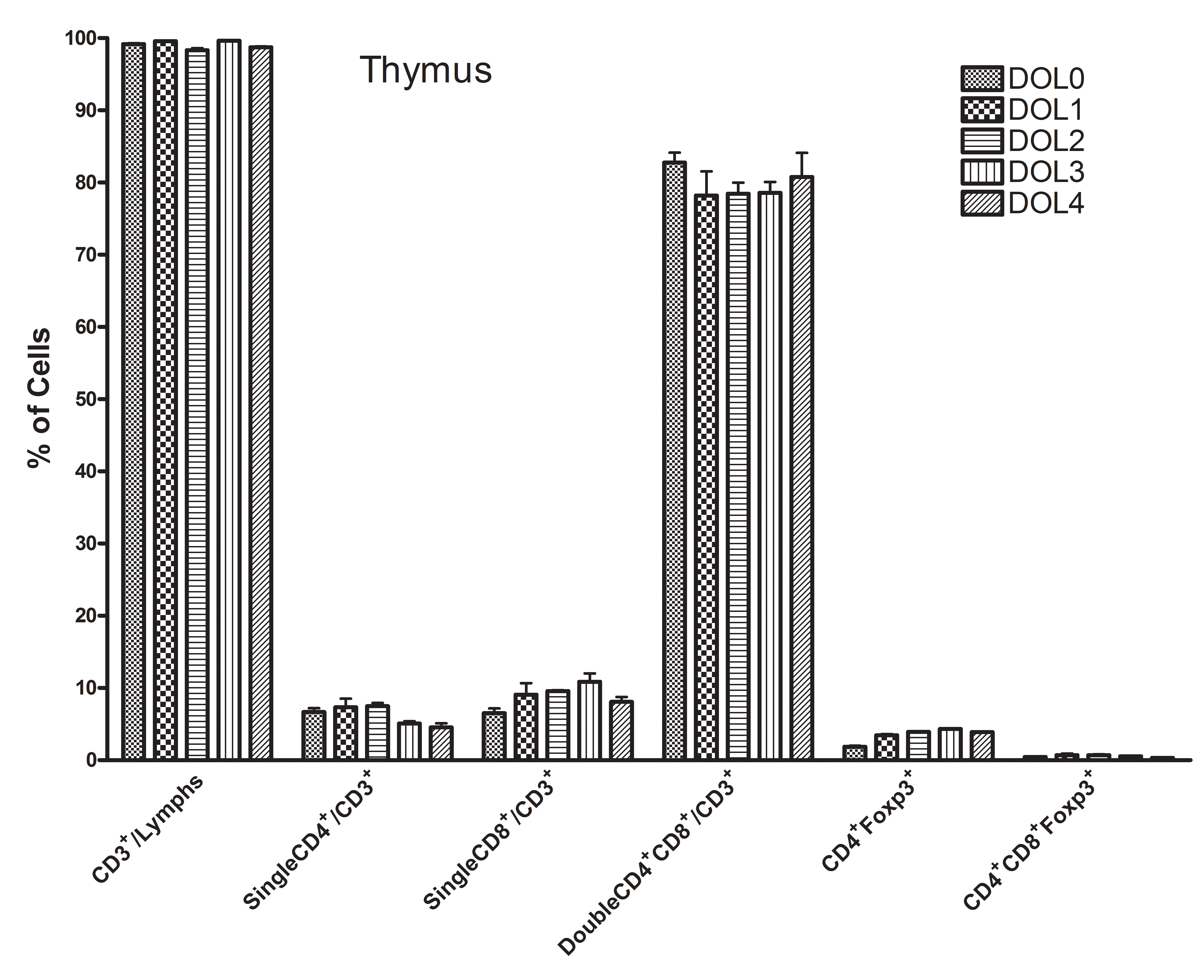

Supplement: Figure S3 — Phenotypic composition of T cell subsets in the thymus of normal rats during DOL 0 to DOL 4. The bars reflect the percentage of cells, shown as means ± SE, N = 9 pups at each DOL. There were no significant differences between DOLs 1, 2, 3, or 4 compared with DOL 0. Thymocytes are >80% CD3+CD4+CD8+ T cells. (TIF) [file pone.0056547.s003.tif]
